# Repurposing Sigma-1 Receptor Ligands for COVID-19 Therapy?

**DOI:** 10.3389/fphar.2020.582310

**Published:** 2020-11-09

**Authors:** José Miguel Vela

**Affiliations:** Drug Discovery and Preclinical Development, ESTEVE Pharmaceuticals, Barcelona, Spain

**Keywords:** COVID-19, SARS-CoV-2, anti-viral, repurposed drugs, drug repurposing, sigma-1 receptor, ER stress, viral replication

## Abstract

Outbreaks of emerging infections, such as COVID-19 pandemic especially, confront health professionals with the unique challenge of treating patients. With no time to discover new drugs, repurposing of approved drugs or in clinical development is likely the only solution. Replication of coronaviruses (CoVs) occurs in a modified membranous compartment derived from the endoplasmic reticulum (ER), causes host cell ER stress and activates pathways to facilitate adaptation of the host cell machinery to viral needs. Accordingly, modulation of ER remodeling and ER stress response might be pivotal in elucidating CoV-host interactions and provide a rationale for new therapeutic, host-based antiviral approaches. The sigma-1 receptor (Sig-1R) is a ligand-operated, ER membrane-bound chaperone that acts as an upstream modulator of ER stress and thus a candidate host protein for host-based repurposing approaches to treat COVID-19 patients. Sig-1R ligands are frequently identified in in vitro drug repurposing screens aiming to identify antiviral compounds against CoVs, including severe acute respiratory syndrome CoV-2 (SARS-CoV-2). Sig-1R regulates key mechanisms of the adaptive host cell stress response and takes part in early steps of viral replication. It is enriched in lipid rafts and detergent-resistant ER membranes, where it colocalizes with viral replicase proteins. Indeed, the non-structural SARS-CoV-2 protein Nsp6 interacts with Sig-1R. The activity of Sig-1R ligands against COVID-19 remains to be specifically assessed in clinical trials. This review provides a rationale for targeting Sig-1R as a host-based drug repurposing approach to treat COVID-19 patients. Evidence gained using Sig-1R ligands in unbiased *in vitro* antiviral drug screens and the potential mechanisms underlying the modulatory effect of Sig-1R on the host cell response are discussed. Targeting Sig-1R is not expected to reduce dramatically established viral replication, but it might interfere with early steps of virus-induced host cell reprogramming, aid to slow down the course of infection, prevent the aggravation of the disease and/or allow a time window to mature a protective immune response. Sig-1R-based medicines could provide benefit not only as early intervention, preventive but also as adjuvant therapy.

## Introduction

The newly emerged 2019 novel coronavirus (CoV), named as severe acute respiratory syndrome CoV-2 (SARS-CoV-2), has been associated with high infection rates and has spread rapidly to become a pandemic (COVID-19 pandemic) since its identification in patients with severe pneumonia in Wuhan, China. Unfortunately, no vaccine has yet been approved to treat human CoVs and the discovery and development of new drugs will require years. Accordingly, repurposing of approved drugs or drugs in clinical development has emerged as a feasible approach to reduce the time compared to *de novo* drug discovery and ultimately to provide a faster treatment option for COVID-19 patients.

CoV replication is structurally and functionally associated with the endoplasmic reticulum (ER) (Sola et al., 2015), and CoV infection is well known to activate pathways to facilitate adaptation of ER stress to viral needs. These embrace hijacking the host cell ER stress responses to modulate protein translation, ER protein folding capacity, ER-associated degradation (ERAD), including autophagy, and apoptotic cell death (Fung and Liu, 2014; Fung et al., 2014a). Therefore, modulation of ER stress response might be pivotal in elucidating CoV-host interactions and might provide the rationale for new therapeutic approaches.

The sigma-1 receptor (Sig-1R) acts as an upstream modulator of ER stress. Sig-1R is a ligand-operated, membrane-bound chaperone that normally reside at the ER-mitochondrion contact called the (mitochondrion-associated ER membrane (MAM), where it regulates ER-mitochondrion signaling and ER-nucleus crosstalk (Hayashi, 2019). Mitochondrial function regulation by Sig-1R includes bioenergetics and free radical generation/oxidative stress. When cells undergo stress, Sig-1R translocates from the MAM to the ER reticular network and plasma membrane to regulate a variety of functional proteins. Via its molecular chaperone activity, the Sig-1R regulates protein folding/degradation, calcium (Ca^2+^) homeostasis, ER stress responses, autophagy, and ultimately cell survival (Hayashi and Su, 2007; Su et al., 2010; Schrock et al., 2013; Vollrath et al., 2014; Hayashi, 2019; Delprat et al., 2020). Interestingly, its chaperone activity can be activated or inhibited by synthetic Sig-1R ligands in an agonist-antagonist manner.

As it regards to its potential antiviral activity, Sig-1R ligands are frequently identified in in vitro drug repurposing screens aiming to identify antiviral compounds against SARS-CoV-2 and other CoVs. Mechanistically, Sig-1R is involved in cellular stress pathways which are used by viruses to promote viral replication (Vasallo and Gastaminza, 2015). Accordingly, Sig-1R has been shown to colocalize with viral replicase proteins in membranous compartments (Friesland et al., 2013), and it has been recently reported that the non-structural (NS) SARS-CoV-2 protein Nsp6 directly interacts with Sig-1R (Gordon et al., 2020). Sig-1R is expressed at substantial density in rodent (Lever et al., 2015) and human (Stone et al., 2006) lungs.

Here pharmacological and genetic data supporting a role for Sig-1R in viral infection are collected and summarized, with a focus on CoV in general and SARS-CoV-2 in particular. Targeting Sig-1R is identified as a potential drug repurposing approach to treat COVID-19 patients that, unlike virus-targeted antiviral agents, addresses adaptive cellular mechanisms of host cells that are crucial for viral infection.

## Sigma-1 Receptor Ligands Exert Antiviral Activity

### Pharmacology Findings Against Non-Coronaviruses

The first insight about a potential role for Sig-1R ligands as antivirals was probably published in 1984 (Nemerow and Cooper, 1984). In this study, several phenothiazines, including trifluoperazine, chlorpromazine, prochlorpromazine and promethazine as well haloperidol (non-phenothiazine but butyrophenone) were shown to inhibit infection of B lymphocytes by a human herpesvirus, Epstein-Barr virus (EBV). By this time sigma was just starting to be considered a separate binding site from phencyclidine and mu and delta opioid receptors to which (+)-[^3^H]SKF10,047 binds (Su, 1982; Tam, 1983). Also by this time, different non-selective neuroleptics including haloperidol, trifluoperazine, chlorpromazine and promethazine were shown to bind this sigma site (Su, 1982; Tam and Cook, 1984) (Table 1), but this was twelve years before the Sig-1R was first cloned (Hanner et al., 1996). Accordingly, authors did not mention sigma mechanisms and attributed the antiviral efficacy of these drugs to effects on calmodulin-regulated cellular endocytic processes involved in early stages of EBV infection. These non-selective Sig-1R ligands were found later to exert antiviral activity against other viruses, including coronaviruses (Table 1).

**TABLE 1:** Drug class.

Intended therapeutic effect	Compound	Sigma-1 receptor affinity		Antiviral activity	
		Ki or IC50^a^ (nM)	References	Virus	References
Antiarrhythmic	Amiodarone	1.4–2.1	Moebius et al., 1997	EBOV	(Madrid et al., 2015)
		335^a^	Buschman, 2007/Internal data		(Gehring et al., 2014)
					(Salata et al., 2015)
					(Dyall et al., 2018)
				HCV	(Gastaminza et al., 2010)
					(Chockalingam et al., 2010)
					(Cheng et al., 2013)
				SARS-CoV	(Stadler et al., 2008)
				SARS-CoV-2	(Mirabelli et al., 2020)
Antidepressant Anxiolytic	Amitriptyline	287	Werling et al. 2007	FLUAV (H5N1)	(Huang et al., 2020)
		216^a^	Buschman, 2007/Internal data	HCV	(Chockalingam et al., 2010)
		300^a^	Weber et al., 1986		
Antimalarial	Amodiaquine	355^a^	Buschman, 2007/Internal data	EBOV	(Madrid et al., 2013)
				DENV	(Boonyasuppayakorn et al., 2014)
				MARV	(Madrid et al., 2013)
				MERS-CoV	(Dyall et al., 2014)
				SARS-CoV	(Dyall et al., 2014)
				SARS-CoV-2	(Jeon et al., 2020; Weston et al., 2020)
Antihistaminic	Astemizole	43^a^	Buschman, 2007/Internal data	EBOV	(Johansen et al., 2015)
				MERS-CoV	(Dyall et al., 2014)
				SARS-CoV	(Dyall et al., 2014)
Antiallergic	Azelastine	274^a^	Buschman, 2007/Internal data	HCV	(Gastaminza et al., 2010)
Antitussive	Benproperine	19^a^	Buschman, 2007/Internal data	HCV	(Gastaminza et al., 2010)
Antiparkinsonian Treatment dystonia and extrapyramidal side effects of antipsychotics	Benztropine	65^a^	Buschman, 2007/Internal data	EBOV	(Madrid et al., 2015)
					(Johansen et al., 2015)
				HCV	(Chockalingam et al., 2010)
					(Mingorance et al., 2014)
				MERS-CoV	(Dyall et al., 2014)
				SARS-CoV SARS-CoV-2	(Dyall et al., 2014; Weston et al., 2020)
Antiarrhythmic and antianginal	Bepridil	365^a^	Buschman, 2007/Internal data	EBOV	(Johansen et al., 2015)
Antihypertensive	Carvedilol	1570^a^	Buschman, 2007/Internal data	HCV	(Gastaminza et al., 2010)
Serotonergic	CGS 12066B	1180^a^	Buschman, 2007/Internal data	HCV	(Gastaminza et al., 2010)
Antimalarial	Chloroquine	108.6	PDSP Ki Database certified data	CCHFV	(Ferraris et al., 2015)
		2300^a^	Buschman, 2007/Internal data	CHIKV	(Bassetto et al., 2013)
					(Pohjala et al., 2011)
				DENV	(Farias et al., 2013)
				EBOV	(Madrid et al., 2013)
					(Madrid et al., 2015)
				FLUAV (H1N1 and H3N2)	(Ooi et al., 2006)
				FLUAV (H5N1)	(Yan et al., 2013)
				HCoV-229E	(de Wilde et al., 2014)
				HCoV-OC43	(Keyaerts et al., 2009)
				HIV-1	(Savarino et al., 2001)
				MARV	(Madrid et al., 2013)
				MERS-CoV	(de Wilde et al., 2014)
					(Dyall et al., 2014)
				SARS-CoV	(de Wilde et al., 2014)
					(Keyaerts et al., 2004)
					(Dyall et al., 2014)
				SARS-CoV-2	(Yao et al., 2020)
					(Jeon et al., 2020; Weston et al., 2020)
Antihistaminic Antiparkinsonian	Chlorphenoxamine	1760^a^	Buschman, 2007/Internal data	MERS-CoV	(Dyall et al., 2014)
				SARS-CoV	(Dyall et al., 2014)
Antipsychotic	Chlorpromazine	146	Tam and Cook, 1984	CCHFV	(Ferraris et al., 2015)
		200^a^	Lang et al., 1994	CHIKV	(Pohjala et al., 2011)
		1070^a^	Buschman, 2007/Internal data	EBV	(Nemerow and Cooper, 1984)
				FLUAV	(Nugent and Shanley, 1984)
				HCoV-229E	(de Wilde et al., 2014)
				HCV	(Mingorance et al., 2014)
				MERS-CoV	(Dyall et al., 2014)
					(de Wilde et al., 2014)
				SARS-CoVSARS-CoV-2	(Dyall et al., 2014)
					(Jeon et al., 2020; Weston et al., 2020)
Antihistaminic Antivertigo	Cinnarizine	22	Klein and Musacchio, 1989	HCV	(Chockalingam et al., 2010)
		119^a^	Buschman, 2007/Internal data		
Antihistaminic	Clemastine	67	Gregori-Puigjané et al., 2012	EBOV	(Johansen et al., 2015)
		505^a^	Buschman, 2007/Internal data	SARS-CoV-2	(Gordon et al., 2020)
Neuroprotectant	Clobenpropit	1080^a^	Buschman, 2007/Internal data	HCV	(Gastaminza et al., 2010)
Estrogen receptor modulator	Clomiphene	4.7–12	Moebius et al., 1997	EBOV	(Madrid et al., 2015)
Ovulation stimulator		195^a^	Buschman, 2007/Internal data		(Johansen et al., 2013; Johansen et al., 2015)
				HCV	(Murakami et al., 2013)
					(Mingorance et al., 2014)
Antidepressant	Clomipramine	195^a^	Buschman, 2007/Internal data	EBOV	(Johansen et al., 2015)
				HCV	(Mingorance et al., 2014)
				MERS-CoV	(Dyall et al., 2014)
				SARS-CoV SARS-CoV-2	(Dyall et al., 2014; Weston et al., 2020)
Antihistaminic Antitussive	Cloperastine	20	Gregori-Puigjané et al., 2012	SARS-CoV-2	(Gordon et al., 2020)
		277^a^	Buschman, 2007/Internal data		
Antifungal	Cycloheximide	1030^a^	Buschman, 2007/Internal data	MERS-CoV	(Dyall et al., 2014
				SARS-CoV	(Dyall et al., 2014
Antiallergic Antihistaminic	Cyproheptadine	284^a^	Buschman, 2007/Internal data	HCV	(Gastaminza et al., 2010)
		930	He et al., 2012		(Chockalingam et al., 2010)
Antidepressant	Desipramine	1190^a^	Buschman, 2007/Internal data	HCV	(Mingorance et al., 2014)
		1987	Narita et al., 1996		
Antiallergic	Desloratadine	1510^a^	Buschman, 2007/Internal data	HCV	(Gastaminza et al., 2010)
Antidepressant	Doxepin	394^a^	Buschman, 2007/Internal data	CHIKV	(Pohjala et al., 2011)
				HCV	(Gastaminza et al., 2010)
Selective sigma-1 receptor antagonist	E-52862 (S1RA)	17	Romero et al., 2012	SARS-CoV-2	(Mirabelli et al., 2020)
Antimigraine	Flunarizine	28^a^	Buschman, 2007/Internal data	HCV	(Chockalingam et al., 2010)
Antidepressant	Fluoxetine	240	Narita et al., 1996	HCV	(Mingorance et al., 2014)
		949^a^	Buschman, 2007/Internal data	HCV	
Antipsychotic	Flupentixol	70^a^	Buschman, 2007/Internal data	EBOV	(Johansen et al., 2015)
Antipsychotic	Fluphenazine	13	Tam and Cook, 1984	EBOV	(Johansen et al., 2015)
		62	Largent et al., 1984	HCV	(Gastaminza et al., 2010)
		109^a^	Buschman, 2007/Internal data	MERS-CoV	(Chockalingam et al., 2010)
					(Dyall et al., 2014)
				SARS-CoV SARS-CoV-2	(Dyall et al., 2014; Weston et al., 2020)
Antipsychotic	Fluspirilene	150	Schotte et al., 1996	MERS-CoV	(Dyall et al., 2014)
		380^a^	Contreras et al., 1990	SARS-CoV	(Dyall et al., 2014)
		563^a^	Buschman, 2007/Internal data	SARS-CoV-2	(Weston et al., 2020)
Antipsychotic	Haloperidol	0.2	Hanner et al., 1996	EBV	(Nemerow and Cooper, 1984)
		1.1	Schotte et al., 1996	HCV	(Gastaminza et al., 2010)
		1.1^a^	Lang et al., 1994	MERS-CoV	(Dyall et al., 2014)
		1.2	Akunne et al., 1997	SARS-CoV	(Dyall et al., 2014)
		2.4	Largent et al., 1984	SARS-CoV-2	(Gordon et al., 2020)
		3	Tam and Cook, 1984		
		6.5–7.3	Su, 1991		
		17^a^	Weber et al., 1986		
		73^a^	Buschman, 2007/Internal data		
Antimalarial	Hydroxychloroquine	2120^a^	Buschman, 2007/Internal data	DENV	(Wang et al., 2015)
				HIV-1	(Sperber et al., 1993)
				MERS-CoV	(Dyall et al., 2014)
				SARS-CoV	
				SARS-CoV-2	(Gordon et al., 2020)
					(Yao et al., 2020)
					(Mirabelli et al., 2020; Weston et al., 2020)
Antiallergic	Hydroxyzine	342^a^	Buschman, 2007/Internal data	HCV	(Mingorance et al., 201)4
Antihistaminic		46	Klein and Musacchio, 1989		
Anxiolytic					
Neuroprotectant Anticonvulsant Antihypertensive	Ifenprodil	1.02	Gitto et al., 2014	FLUAV (H5N1)	(Zhang et al., 2019)
		2–2	Hanner et al., 1996	HCV	(Chockalingam et al., 2010)
		5.5^a^	Buschman, 2007/Internal data		
		28–34	Su, 1991		
Antidepressant	Imipramine	343	Narita et al., 1996	HCV	(Mingorance et al., 2014)
		529^a^	Buschman, 2007/Internal data		
Antiadictive	Indatraline	737^a^	Buschman, 2007/Internal data	HCV	(Gastaminza et al., 2010)
Antihistaminic	Ketotifen	3800^a^	Buschman, 2007/Internal data	HCV	Gastaminza et al., 2010)
Antidepressant	Lofepramine	2520	PDSP Ki Database certified data	HCV	(Gastaminza et al., 2010)
		100% inh. at 10 *µ*M	Buschman, 2007/Internal data		
Antimigraine	Lomerizine	37^a^	Buschman, 2007/Internal data	EBOV	(Johansen et al., 2015)
Antidiarrheal	Loperamide	271	Buschman, 2007/Internal data	HCoV-229E	(de Wilde et al., 2014)
				MERS-CoV	
				SARS-CoV	
				SARS-CoV-2	(Jeon et al., 2020)
Antidepressant	Maprotiline	37^a^	Buschman, 2007/Internal data	EBOV	(Johansen et al., 2015)
Antimalarial	Mefloquine	2560^a^	Buschman, 2007/Internal data	JCV	(Brickelmaier et al., 2009)
				MERS-CoV	(Dyall et al., 2014)
				SARS-CoV	(Dyall et al., 2014)
				SARS-CoV-2	(Jeon et al., 2020; Weston et al., 2020)
Antihistaminic	Methdilazine	167^a^	Buschman, 2007/Internal data	CHIKV	(Pohjala et al., 2011)
Sigma ligand	PB28	0.38	Abate et al., 2011	SARS-CoV-2	(Gordon et al., 2020)
		10–13	Azzariti et al., 2006		
Sigma ligand	PD-144418	0.08	Akunne et al., 1997	SARS-CoV-2	(Gordon et al., 2020)
		0.46	Lever et al., 2014		
Antipsychotic Antiemetic Anxiolytic	Perphenazine	8	Tam and Cook, 1984	CHIKV	(Pohjala et al., 2011
		13	Largent et al., 1984	HCV	(Chockalingam et al., 2010)
		21^a^	Weber et al., 1986		(Mingorance et al., 2014)
		45–53	Su, 1991		
		104^a^	Buschman, 2007/Internal data		
Antipsychotic Treatment of Tourette syndrome and resistant tics	Pimozide	139	Tam and Cook, 1984	EBOV	(Johansen et al., 2015)
		508	Largent et al., 1984	HCV	(Chockalingam et al., 2010)
		337^a^	Buschman, 2007/Internal data		
Antipsychotic	Piperacetazine	823^a^	Buschman, 2007/Internal data	EBOV	(Johansen et al., 2015)
Antipsychotic Antiemetic Anxiolytic	Prochlorperazine	232^a^	Buschman, 2007/Internal data	EBOV	(Madrid et al., 2015)
					(Johansen et al., 2015)
				HCV	(Gastaminza et al., 2010)
					(Chockalingam et al., 2010)
Endogenous steroid Menopausal hormone therapy	Progesterone	173–196	Su, 1991	SARS-CoV-2	(Gordon et al., 2020)
		1960^a^	Buschman, 2007/Internal data		
		260–338	Hanner et al., 1996		
Antiallergic Antihistaminic	Promethazine	857^a^	Buschman, 2007/Internal data	EBV	(Nemerow and Cooper, 1984)
				MERS-CoV	(Dyall et al., 2014)
				SARS-CoV SARS-CoV-2	(Dyall et al., 2014; Weston et al., 2020)
Antidepressant	Protriptyline	307^a^	Buschman, 2007/Internal data	HCV	(Chockalingam et al., 2010)
Antimalarial	Quinacrine	953^a^	Buschman, 2007/Internal data	EBOV	(Johansen et al., 2015)
Antiarrhythmic	Quinidine	570	Musacchio and Klein, 1988	HCV	(Chockalingam et al., 2010)
		5480^a^	Buschman, 2007/Internal data		
Serotonergic	Quipazine-6N	1250^a^	Buschman, 2007/Internal data	HCV	(Chockalingam et al., 2010)
Estrogen receptor modulator Treatment of osteoporosis in postmenopausal women	Raloxifene	38	Laggner et al., 2005	HCV	(Chockalingam et al., 2010)
		122^a^	Buschman, 2007/Internal data		(Mingorance et al., 2014)
					(Murakami et al., 2013)
Sigma ligand	Rimcazole	260^a^	Lang et al., 1994	HCV	(Gastaminza et al., 2010)
		500^a^	Ferris et al., 1986		
		820	Gilligan et al., 1994		
		908	Husbands et al., 1999		
Antitumoral (breast cancer)	Ritanserin	190^a^	Buschman, 2007/Internal data	HCV	(Chockalingam et al., 2010)
Antiasthmatic	Salmeterol	151^a^	Buschman, 2007/Internal data	HCV	(Gastaminza et al., 2010)
Antidepressant Anxiolytic	Sertraline	57	Narita et al., 1996	EBOV	(Johansen et al., 2015)
		260^a^	Buschman, 2007/Internal data		
Sigma ligand	Siramesine	17^a^	Perregaard et al., 1995	SARS-CoV-2	(Gordon et al., 2020)
Estrogen receptor modulator	Tamoxifen	34–26	Moebius et al., 1997	HCV	(Mingorance et al., 2014)
Antitumoral (breast cancer)		367^a^	Buschman, 2007/Internal data	HSV-1	(Murakami et al., 2013)
					(Zheng et al., 2014)
				MERS-CoV	(Dyall et al., 2014)
				SARS-CoV SARS-CoV-2	(Dyall et al., 2014; Weston et al., 2020)
Antifungal	Terconazole	159^a^	Buschman, 2007/Internal data	EBOV	(Johansen et al., 2015)
				MERS-CoV	(Dyall et al., 2014)
				SARS-CoV SARS-CoV-2	(Dyall et al., 2014; Weston et al., 2020)
Antiemetic	Thiethylperazine	528^a^	Buschman, 2007/Internal data	CHIKV	(Pohjala et al., 2011)
				MERS-CoV	(Dyall et al., 2014)
				SARS-CoV SARS-CoV-2	(Dyall et al., 2014; Weston et al., 2020)
Antipsychotic	Thioridazine	286^a^	Buschman, 2007/Internal data	CHIKV	(Pohjala et al., 2011)
				EBOV	(Johansen et al., 2015)
Antipsychotic	Thiothixene	353^a^	Buschman, 2007/Internal data	MERS-CoV	(Dyall et al., 2014)
				SARS-CoV	(Dyall et al., 2014)
Estrogen receptor modulator	Toremifene	220^a^	Buschman, 2007/Internal data	EBOV	(Johansen et al., 2013; Johansen et al., 2015)
Antitumoral (breast cancer)				HCV	(Gastaminza et al., 2010)
				MERS-CoV	(Dyall et al., 2014)
				SARS-CoV SARS-CoV-2	(Dyall et al., 2014; Weston et al., 2020)
Antipsychotic	Trifluoperazine	15–21	Hanner et al., 1996	EBV	(Nemerow and Cooper, 1984)
		54	Tam and Cook, 1984	HCV	(Gastaminza et al., 2010)
		125^a^	Buschman, 2007/Internal data		(Chockalingam et al., 2010)
		345^a^	Weber et al., 1986		
Antipsychotic Antiemetic	Triflupromazine	154	Tam and Cook, 1984	HCV	(Chockalingam et al., 2010)
		470^a^	Buschman, 2007/Internal data	MERS-CoV	(Dyall et al., 2014)
		605^a^	Weber et al., 1986	SARS-CoV	(Dyall et al., 2014)
Antihypertensive	Verapamil	258^a^	Buschman, 2007/Internal data	FLUAV	(Nugent and Shanley, 1984)
Antiarrhythmic				SARS-CoV-2	(Mirabelli et al., 2020)
Antipsychotic	(−)-Butaclamol	40	Tam and Cook, 1984	HCV	(Gastaminza et al., 2010)
		95.7	Akunne et al., 1997		
		157	Largent et al., 1984		
		183^a^	Weber et al., 1986		
Antipsychotic	(±)-Butaclamol	343^a^	Buschman, 2007/Internal data	HCV	(Gastaminza et al., 2010)

^a^IC50. CHIKV, Chikungunya virus; HCV, Hepatitis C virus; FLUAV, Influenza A virus; H5N1, Avian influenza A H5N1 virus, other subtypes; CCHFV, Crimean-Congo hemorrhagic virus; HSV-1, Herpes simplex virus type 1; JCV, JC (John Cunningham) virus; DENV, Dengue virus; HCoV-229E, Human coronavirus strain 229E; MARV, Marburg hemorrhagic fever virus; MERS-CoV, East respiratory syndrome coronavirus; SARS-CoV, Human coronavirus strain OC43 (HCoV-OC43) Severe acute respiratory syndrome coronavirus; EBOV, Ebola virus; HIV-1, Human immunodeficiency virus type 1; SARS-CoV-2, Severe acute respiratory syndrome coronavirus type 2; EBV, Epstein-Barr virus.

Drugs binding Sig-1R showing antiviral activity were identified in three *in vitro* screening studies aiming to discover inhibitors targeting different steps of Hepatitis C virus (HCV) infection. In the first study, a set of 446 compounds from the National Institutes of Health Clinical Collection were assayed for their ability to inhibit HCV infection of human hepatocarcinoma Huh-7 cells *in vitro* (Gastaminza et al., 2010). Compounds were screened in a cell-based assay in an unbiased manner, independent on target specificity or mechanism of action. Among the 446 clinically approved small molecules assayed, 33 compounds displayed antiviral activity (>85% reduction in HCV infection of Huh-7 cells, as compared to the vehicle DMSO control) in the absence of cytotoxicity at low micromolar and submicromolar concentrations. Compounds targeted several aspects of HCV infection, including entry, replication, and assembly. Some of the active antiviral compounds were already known to have antiviral activity, but the ability of most of them to inhibit HCV infection was unexpected. Among the 33 active compounds, 19 compounds (cyproheptadine, toremifene, fluphenazine, trifluoperazine, CGS 12066B, prochlorperazine, doxepin, ketotifen, amiodarone, lofepramine, rimcazole, clobenpropit, salmeterol, azelastine, desloratadine, indatraline, haloperidol, benproperine and carvedilol) bind to Sig-1R with high to moderate affinity (Table 1). All of them are non-selective and bind primarily to molecular targets other than Sig-1R, but it is remarkable that near 60% of active compounds inhibiting >85% HCV infection had known affinity for Sig-1R. Most of these compounds inhibited HCV entry and display selective anti-HCV activity relative to vesicular stomatitis virus (VSV)-pseudotypes. In another unbiased cell-based screening, a chemical library of 281 clinically approved drugs prescribed for non-HCV applications were assayed. Twelve compounds reduced HCV infection by more than one order of magnitude without significantly reducing cell biomass (Mingorance et al., 2014). Surprisingly, all of them (chlorpromazine, clomipramine, desipramine, perphenazine, imipramine, raloxifene, tamoxifen, clomiphene, hydroxyzine, benztropine and fluoxetine) bind to Sig-1R with significant affinity (Table 1). Hydroxyzine and benztropine were selected to define the step of the replication cycle they target. Both HCV inhibitors interfered with an early step of the infection, at a step downstream viral particle attachment and internalization but previous to the establishment of persistent RNA replication and infectious virus production. Together, results reinforced the notion that compounds inhibit an early step of HCV RNA replication. The involvement of Sig-1R was not discussed in these papers, but authors noted that affinity for this molecular target was shared by a significant number of active compounds, which evoked studies addressing specifically the role of Sig-1R in HCV infection (Friesland et al., 2013).

In the third of these screening studies, 1280 compounds, many in clinical trials or approved for therapeutic use, were assayed for their ability to alleviate the HCV-induced cytopathic effect on the engineered cell line n4mBid (Chockalingam et al., 2010). They found >200 hits able to increase n4mBid cell viability relative to untreated cells. Of the 55 leading hits, 47 compounds inhibited one or more aspects of the HCV life cycle (entry, replication or infectious virus assembly/release) by >40%. Interestingly, significant affinity for the Sig-1R has been reported for 19 of them: amiodarone, amitriptyline, benztropine, butaclamol, cinnarizine, cyproheptadine, flunarizine, fluphenazine, ifenprodil, prochlorperazine, perphenazine, pimozide, protriptyline, quinidine, quipazine-6N, raloxifene, ritanserin, triflupromazine and trifluoperazine (Table 1). Interaction with Sig-1R has also been suggested for biperiden (Yoshida et al., 2000) and SKF-38393 has been described as allosteric modulator of the Sig-1R (Guo et al., 2013). That is, 21 out of 55 leads identified in the cell protection small-molecule screen against HCV were known sigma-1 binders. All of them are non-selective and typically known by their activity on molecular targets other than Sig-1R. Affinity data for Sig-1R are unknown or have not been reported for the rest of identified anti-HCV compounds and thus the possibility that Sig-1R-mediated mechanisms contribute to their effect cannot be ruled-out. Inhibition of entry and infectious virus production, assembly and release accounted for the protective effect of most known Sig-1R ligands, although some of them also inhibited HCV replication. The potential contribution of Sig-1R was not discussed.

Changes in cell death induced by avian influenza A (FLUAV) H5N1 virus in A549 lung epithelial cells were explored using RNA interference (RNAi) screening methods. These screens identified multiple genes for which knockdown altered cell viability and drugs targeting some of these genes were assayed for their potential antiviral activity. The neurological drug ifenprodil increased cell viability *in vitro* and markedly decreased leukocyte infiltration and lung injury, and improved survival of mice infected with H5N1 (Zhang et al., 2019), the most lethal influenza virus strain. The effect of ifenprodil was discussed in the context of its antagonism at the N-methyl-D-aspartate (NMDA) receptor as overstimulation of the NMDA receptor can trigger lung injury. In another study sharing authors with the previous one, genes and pathways differentially expressed in A549 cells upon FLUAV H5N1 virus infection were identified and some drugs were assayed as potential treatments (Huang et al., 2020). Amitriptyline increased viability of A549 cells infected with H5N1 for 48 h when assayed 1 h before infection or at 3 h after infection, and reduced the infiltrating cell count, decreased lung injury, improved lung edema and survival of H5N1 virus-infected mice. The involvement of Sig-1R in mediating the effects of these drugs on influenza A H5N1 virus infection was not discussed, although ifenprodil shows high affinity for Sig-1R (Hashimoto and London, 1995; Gitto et al., 2014). Amitriptyline, a non-selective antidepressant binding to multiple receptors and transporters, also binds to Sig-1R with moderate affinity (Werling et al., 2007). Note that ifenprodil and amitriptyline were previously shown to inhibit the HCV-induced cytopathic effect (Chockalingam et al., 2010).

Regarding filoviruses, a systematic *in vitro* screen of FDA-approved drugs was performed to identify compounds with antiviral activities against the Ebola virus (EBOV) (Madrid et al., 2015). Assays were conducted in the Vero cell line. Active compounds (>50% viral inhibition and <30% cellular toxicity) at a single concentration were tested in dose-response assays. On the basis of the approved human dosing, toxicity/tolerability and pharmacokinetic data, seven *in vitro* hits were selected and evaluated for their *in vivo* efficacy. Five of the seven (chloroquine, amiodarone, prochlorperazine, benztropine, and clomiphene) hit compounds show affinity for the Sig-1R (Table 1), although the contribution of Sig-1R-mediated mechanisms was not discussed in the paper. When administered *in vivo* in a mouse model, azithromycin (100 mg/kg, twice daily, i.p.), chloroquine (90 mg/kg, twice daily, i.p.), and amiodarone (60 mg/kg, twice daily, i.p.) increased survival of infected mice, but only chloroquine gave significant reproducible efficacy with this dosing regimen. Azithromycin and chloroquine were also tested in a guinea pig model of EBOV infection, but none of the tested doses increased survival. In a separate study, also testing FDA-approved drugs (∼2600 drugs and molecular probes) in an *in vitro* infection assay using the type species Zaire EBOV, selective antiviral activity was found for 80 drugs spanning multiple mechanistic classes (Johansen et al., 2015). A set of 30 active compounds was prioritized. A good number of them (17 out of 30: astemizole, benztropine, bepridil, clemastine, clomiphene, clomipramine, flupentixol, fluphenazine, lomerizine, maprotiline, piperacetazine, prochlorperazine, quinacrine, sertraline, terconazole, thioridazine and toremifene) are known to display affinity for Sig-1R and most of them were indeed identified in previous studies with other viruses (Table 1). Interestingly, results in a murine EBOV infection model confirmed the protective ability of several drugs, notably bepridil and sertraline, which both bind Sig-1R with remarkable affinity (Table 1). Viral entry assays indicated that most of these antiviral drugs block a late stage of viral entry.

Finally, inhibition of the cytopathic effect induced by Chikungunya virus and other alphaviruses (Semliki Forest virus and Sindbis virus) was found for chlorpromazine, doxepin, methdilazine, perphenazine, thiethylperazine, thioridazine and chloroquine (Pohjala et al., 2011), all of them non-selective Sig-1R ligands that also exhibit antiviral activity against other viruses (Table 1).

### Pharmacology Findings Against Coronaviruses

SARS-CoV-2 (severe acute respiratory syndrome-related CoV type 2), the causative virus of COVID-19 pandemic, belongs to the broad family of positive-sense single-stranded RNA (+ssRNA) CoV. Other CoV also cause illnesses ranging from common cold to more severe diseases such as Middle East respiratory syndrome (MERS). It is the seventh known CoV to infect people, after 229E, NL63, OC43, HKU1, MERS-CoV, and the original SARS-CoV (Zhu et al., 2020). Phylogenetic analyses revealed conserved evolutionary relationship between SARS-CoV-2 and SARS-CoV (79.7% nucleotide sequence identity) (Zhou et al., 2020).

In this section, data supporting the involvement of Sig-1R and therapeutic potential of Sig-1R ligands against CoV infection is summarized.

Chloroquine and hydroxychloroquine bind to Sig-1R (Table 1). These antimalarial drugs have shown antiviral activity against different viruses (Sperber et al., 1993; Savarino et al., 2001; Madrid et al., 2013; Ferraris et al., 2015; Wang et al., 2015). There was also evidence supporting the efficacy of chloroquine and hydroxychloroquine against other members of the Coronaviridae family before COVID-19 pandemic. Chlroroquine was described to show antiviral activity against human CoV strain OC43 (HCoV-OC43) (Keyaerts et al., 2009). HCoV-OC43 together with HCoV-229E are responsible for 10 to 30% of all common colds, and infections occur mainly during the winter and early spring (Larson et al., 1980). Chloroquine inhibited HCoV-OC43 replication in HRT-18 cells and prevented HCoV-OC43-induced death in newborn mice when mothers were treated daily with chloroquine (15 mg/kg). On these bases, authors suggested that chloroquine might be considered as a future drug against HCoVs (Keyaerts et al., 2009). Indeed, chloroquine also inhibited the replication of SARS-CoV *in vitro* (Keyaerts et al., 2004) and a number of subsequent studies have confirmed its antiviral activity against SARS-CoV (de Wilde et al., 2014; Dyall et al., 2014) and recently against SARS-CoV-2 (Jeon et al., 2020; Yao et al., 2020).

Chloroquine and its hydroxy analog were by far the most popular drugs proposed initially for treatment and prophylaxis of COVID-19: 208 interventional clinical trials registered on the NIH site involve treatment with these drugs, alone or in combination (ClinicalTrials.gov query, 2020). *In vitro*, both drugs inhibit SARS-CoV-2 infection in Vero cells, but hydroxychloroquine (EC_50_ = 0.72 *μ*M) is more potent than chloroquine (EC_50_ = 5.47 *μ*M) (Yao et al., 2020). The benefits of this treatment have been investigated during the course of this pandemic, yet no scientific evidence supports the widespread use of these medications. In fact, results of the first clinical studies evaluating the effect of hydroxychloroquine do not support efficacy of this drug in COVID-19 patients (Geleris et al., 2020; Mitjà et al., 2020; Roustit et al., 2020). Yet, preliminary studies aroused considerable media interest, raising fears of massive and uncontrolled use of these drugs, inexpensively produced in several countries. On the other hand, serious adverse drug reactions have been reported in patients with COVID-19 receiving hydroxychloroquine. Side effects of both antimalarial drugs are well established, including serious retinopathies and cardiopathies associated with bioaccumulation of the drugs (Palmeira et al., 2020). Recently (June 15, 2020), FDA has revoked the emergency use authorization to use hydroxychloroquine and chloroquine to treat COVID-19 based on findings from a large, randomized clinical trial in hospitalized patients showing no benefit for decreasing the likelihood of death or speeding recovery (FDA communication, 2020). The mechanism of action of these aminoquinolines is thought to depend on their capacity to increase the endosomal pH to inhibit lysosomal enzymes. This prevents enveloped viruses from entering and releasing their genetic material into the host cells (Tripathy et al., 2020). Binding to a ganglioside-binding domain at the N-terminal domain of the SARS-CoV-2 S protein has also been suggested as a mechanism of chloroquine and hydroxychloroquine to inhibit attachment of the virus to lipid rafts and contact with the ACE-2 receptor for entry (Fantini et al., 2020). The only reference to Sig-1R comes from an unrelated study describing protection by chloroquine against glutamate-induced cell death through a Sig-1R-mediated mechanism (Hirata et al., 2011). The eventual contribution of Sig-1R to the antiviral effects of chloroquine and hydroxychloroquine is just starting to be recognized (Gordon et al., 2020; Mirabelli et al., 2020), but they are non-selective Sig-1R ligands and their affinities for this molecular target are suboptimal.

The antiarrhythmic amiodarone, a non-selective but high affinity sigma-1 ligand, was reported to inhibit the spreading *in vitro* of SARS-CoV in Vero cells (Stadler et al., 2008). Amiodarone reduced the virus titer in a concentration-dependent manner, at concentrations at which it has no effect on cell viability. Direct interaction with the SARS-CoV or impairment of virus entry did not account for its antiviral activity, but amiodarone interfered with the SARS-CoV life cycle after delivery of its genome in the cytosol. As a cationic amphiphilic drug, amiodarone (and its main metabolite MDEA) accumulates into late endosomes/lysosomes and reduces their lumenal acidity, precluding acidic cleavage of viral proteins and interfering with the endocytic pathway (Salata et al., 2015). However, amiodarone displayed antiviral activity even when SARS-CoV has delivered its genome into the cytoplasm, thus involving additional mechanisms at a post-endosomal level (Stadler et al., 2008). The contribution (or not) of sigma-1-mediated mechanisms to the antiviral activity of amiodarone was not discussed in the publications. Amiodarone was also shown to inhibit the HCV-induced cytopathic effect on the engineered cell line n4mBid (Chockalingam et al., 2010), HCV entry and assembly steps in Huh-7.5.1 cells (Cheng et al., 2013), and EBOV cell entry in a variety of cultured cell lines (Gehring et al., 2014; Salata et al., 2015; Dyall et al., 2018). Despite promising *in vitro* results, amiodarone failed to protect guinea pigs from a lethal dose of EBOV (Dyall et al., 2018). In the clinical setting, in December 2014, approximately 80 patients in Ebola treatment units in Freetown, Sierra Leone, received amiodarone as a compassionate therapy at doses up to 30 mg/kg per day (ClinicalTrials.gov Identifier NCT02307591, 2014). A decrease in case fatality rate was reported when compared with local historical data. Unfortunately, the study was not a formal clinical trial, and the statistical significance of this result is not known (Turone, 2014; Gupta-Wright et al., 2015). Recently, the case of a patient affected by COVID-19-related respiratory failure who recovered after only supportive measures and off-label short therapy with amiodarone (starting on the second day from admission and lasting 5 days; administered on day 1 as a 15 mg/kg/24 h intravenous infusion, followed by oral administration of 400 mg twice daily) has been reported (Castaldo et al., 2020). Accordingly, amiodarone, widely prescribed to treat both ventricular and supraventricular arrhythmias, has been proposed as a possible therapy (alone or as part of a combination regimen) to prevent SARS-CoV-2 infection rather than to treat symptomatic or severe COVID-19 patients (Aimo et al., 2020; Sanchis-Gomar et al., 2020).

A set of 348 FDA-approved drugs was screened in cell cultures infected with MERS-CoV (de Wilde et al., 2014). Four compounds (chloroquine, chlorpromazine, loperamide, and lopinavir) inhibited MERS-CoV replication in the low-micromolar range (IC_50s_ 3 to 8 *μ*M). These compounds also inhibited the replication of SARS-CoV and HCoV-229E. Interestingly, chloroquine but also chlorpromazine and loperamide bind to Sig-1R (Table 1). Time-of-addition experiments suggested that chloroquine, chlorpromazine and loperamide inhibit an early step in the replicative cycle whereas lopinavir inhibits a post-entry step. This finding is congruent with previous findings showing that Sig-1R regulates early stages of HCV RNA replication (Friesland et al., 2013).

In another study, a library of 290 compounds with FDA approval or in advanced clinical development was screened for antiviral activity against MERS-CoV and SARS-CoV (Dyall et al., 2014). Twenty seven compounds displayed *in vitro* activity against both MERS-CoV and SARS-CoV. Among the 27 active compounds, at least 19 bind with significant affinity to Sig-1R (chloroquine, hydroxychloroquine, mefloquine, amodiaquine, tamoxifen, toremifene, terconazole, cycloheximide, benztropine, fluspirilene, thiothixene, fluphenazine, promethazine, astemizole, chlorphenoxamine, chlorpromazine, thiethylperazine, triflupromazine and clomipramine) (Table 1), though their antiviral activity was not discussed to be related to Sig-1R. Recently, authors prioritized 20 drugs from this previous screening and found that 17 of the 20 tested drugs that inhibited SARS-CoV and MERS-CoV also inhibited the cytopathic effect of SARS-CoV-2 on Vero E6 cells, with similar IC_50_ values and at non-cytotoxic concentrations (Weston et al., 2020). All (amodiaquine, benztropine, chloroquine, chlorpromazine, clomipramine, fluphenazine, fluspirilene, hydroxychloroquine, mefloquine, promethazine, tamoxifen, terconazole, thiethylperazine and toremifene) but two are known to bind Sig-1R (Table 1). Two of them, chloroquine and chlorpromazine, were evaluated *in vivo* using a mouse-adapted SARS-CoV model. Drug treatments did not inhibit virus replication in lungs, but did protect mice from clinical disease (Weston et al., 2020). Note that repurposing not only of chloroquine but also of the antipsychotic chlorpromazine has been proposed to treat COVID-19 (Nobile et al., 2020; Plaze et al., 2020).

In a recent repositioning study, 48 FDA-approved drugs, including 35 drugs pre-selected by their activity against SARS-CoV snd 13 drugs recommended from infectious diseases specialists, were assayed for their antiviral activity against SARS-CoV-2 in Vero cells (Jeon et al., 2020). Infected cells were analyzed by immunofluorescence using an antibody against the viral N protein of SARS-CoV-2. Among the 48 drugs evaluated, 24 showed potential anti-SARS-CoV-2 activity, with IC_50_ values between 0.1 and 10 *µ*M. Three of them, loperamide, mefloquine and amodiaquine, in addition to chloroquine, are known to bind Sig-1R (Table 1). All of them were previously shown to be effective against other CoV, including MERS-CoV and SARS-CoV (Dyall et al., 2014).

In a recent paper, targeting Sig-1R was highlighted based on findings of a SARS-CoV-2 protein interaction map and pharmacological data (Gordon et al., 2020). Screening a subset of drugs identified two sets of pharmacological agents effectively reducing SARS-CoV-2 infectivity in Vero-6 cells: inhibitors of mRNA translation and predicted regulators of the sigma-1 and sigma-2 receptors. Non-selective Sig-1R ligands including haloperidol, PB28, PD-144418 and hydroxychloroquine, and subsequently clemastine, cloperastine, progesterone and siramesine (Table 1) were found to exert antiviral effects. Hydroxychloroquine was among the less potent antiviral of the assayed Sig-1R ligands, which correlated with its lower affinity for this molecular target. Authors discussed the involvement of sigma receptors. They noted that these molecules are also active against other receptors, but the only shared among all of them are the sigma receptors. For instance, the antipsychotic haloperidol inhibits the dopamine D2 and histamine H1 receptors, while clemastine and cloperastine are themselves antihistamines, but all three molecules are Sig-1R ligands and exert antiviral activity. In contrast, the antipsychotic olanzapine, which also inhibits H1 and D2 receptors, has no significant Sig-1R activity and is not antiviral. Authors also noted that the widely used antitussive dextromethorphan exerted proviral activity and stated that its use should merit caution and further study in the context of COVID-19. Dextromethorphan but also carbetapentane, another commonly used antitussive (Brown et al., 2004), the narcotic analgesic pentazocine (particularly its active (+)-pentazocine enantiomer) (Tam and Cook, 1984) and some antidepressants (Narita et al., 1996), among some other marketed compounds, are considered prototype Sig-1R agonists/positive modulators. Thus, should caution be extended to the use of other potential, although non-selective Sig-1R agonists? In this way, cocaine is a non-selective Sig-1R agonist and exposure to cocaine has been shown to enhance HIV infection by activating Sig-1R (Roth et al., 2005). Cocaine use/abuse could thus be a risk factor but, to my knowledge, the effect of cocaine on CoV infections has not been investigated.

Finally, in a recent publication, quantitative high-content morphological profiling coupled with an AI-based machine learning strategy was applied to identify efficacious single agents against SARS-CoV-2 (Mirabelli et al., 2020). This assay detected multiple antiviral mechanisms of action, including inhibition of viral entry, propagation, and modulation of host cellular responses. Viral growth kinetics were assayed at a multiplicity of infection of 0.2 in Huh-7 cells, with peak viral titers at 48 h post infection. From a library of 1,441 FDA-approved compounds and clinical candidates, 15 dose-responsive compounds with antiviral potency below 1 *µ*M and devoid of cytotoxicity were identified. Three of them, amiodarone, verapamil and E-52862 (S1RA) were known to bind Sig-1R (Table 1). Interestingly, E-52862 (S1RA) is a selective Sig-1R antagonist (Romero et al., 2012). It exerted potent activity against SARS-CoV-2 in Huh-7 cells (IC_50_ = 222 nM) and iPSC-derived alveolar epithelial type 2 cells (iAEC2s) (IC_50_ = 1 *µ*M), with limited cell toxicity (CC_50_ > 5000 nM). E-52862 (S1RA) depleted infected cells and induced cellular changes suggestive of a host-modulation mechanism, which led to suggest that the activity of S1RA is dependent on host cell mechanisms (presumably active in Huh-7 and iAEC2s cells but not in Vero-6 cells, which are highly permissive to viral growth) and, promisingly, that human cells may be more responsive to this compound. This (and differences in other experimental conditions) could explain way E-52862 was devoid of activity when assayed in the Vero E6 cell line (Gordon et al., 2020).

## Mechanism of Action

### Sigma-1 Receptor and Viral Entry

Inhibition of viral entry has been reported for non-selective sigma-1 ligands in a number of studies (Chockalingam et al., 2010; Gastaminza et al., 2010; Cheng et al., 2013; Johansen et al., 2015; Dyall et al., 2018), but not in others (Nemerow and Cooper, 1984; Mingorance et al., 2014; Stadler et al., 2008; de Wilde et al., 2014; Gordon et al., 2020). Thus, it is unclear whether prevention of viral particle attachment or internalization accounts for Sig-1R-mediated antiviral effect of such drugs.

Inhibition of HCV entry into Huh-7 human hepatoma cells by sigma-1 ligands was demonstrated in pharmacology studies (Gastaminza et al., 2010), but downregulation of Sig-1R in Huh-7 cells did not affect HCV entry (Friesland et al., 2013). This might suggest that the deficiency of the modulatory sigma-1 protein (as in the case of gene silencing approaches) does not mimic the pharmacological inhibitory effect on viral entry elicited by an antagonist acting at the Sig-1R. Accordingly, absence of the regulatory mechanism in Sig-1R deficient cells would not be equivalent to the inhibitory effect promoted by a Sig-1R ligand on the target protein with which Sig-1R is interacting. This is possible due to the chaperone nature of the Sig-1R, which exerts its action through physical protein–protein interactions (Su et al., 2010; Pabba, 2013).

**FIGURE 1:**
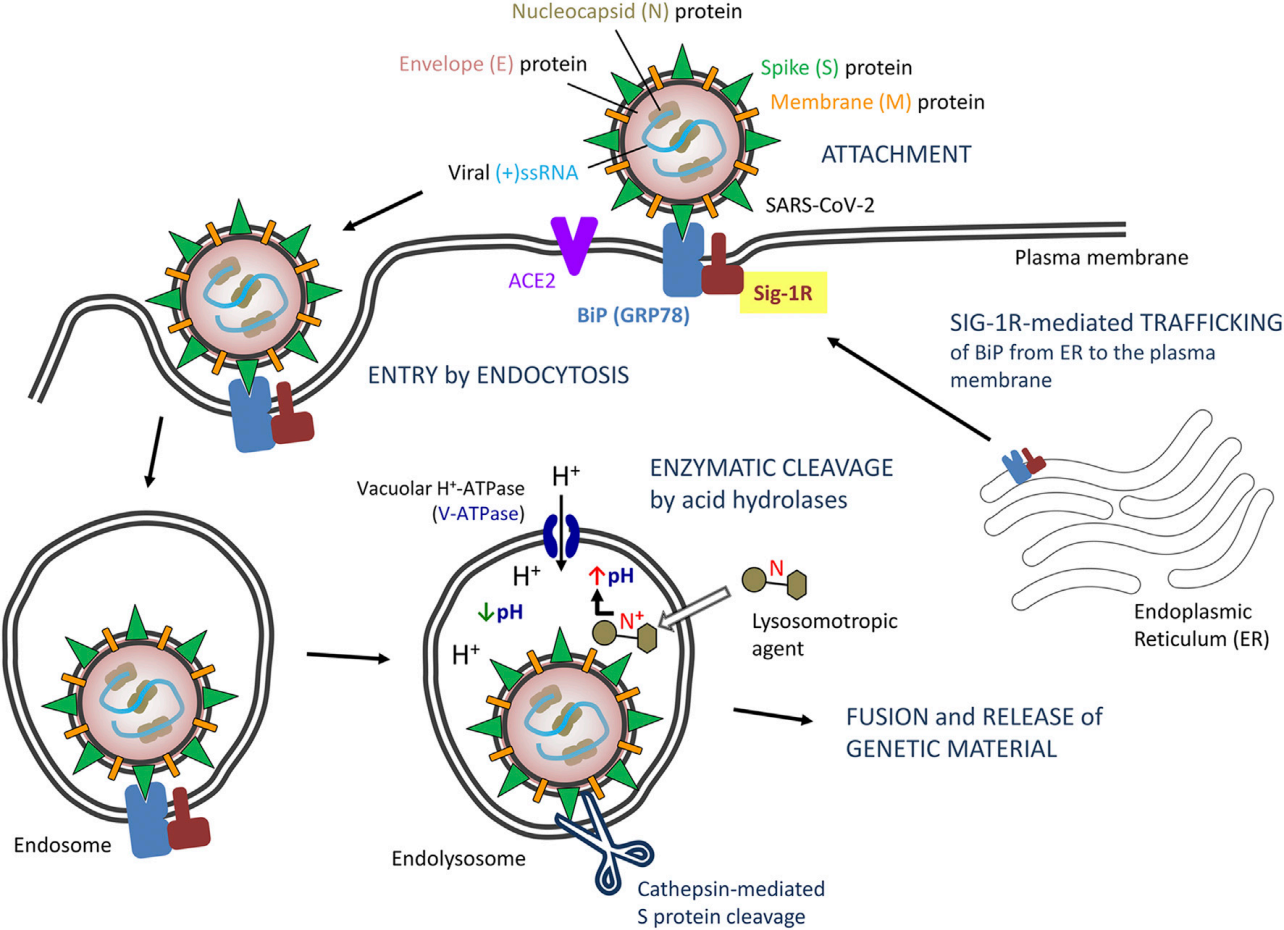
Proposed model of severe acute respiratory syndrome CoV-2 (SARS-CoV-2) entry by endocytosis: Potential role of Sigma-1 receptors (Sig-1Rs) via BiP interaction. Abbreviations and bibliographic references for the role of Sig-1R are provided in the text.

Sig-1R normally resides at the ER, typically at the MAM, but when cells undergo stress (as expected following viral infection) the Sig-1R translocates to the peripheral ER network and plasma membrane to regulate a variety of cell surface proteins (Su et al., 2010), which might account for ligand-operated, Sig-1R-mediated modulation of virus attachment or entry (Figure 1). In this way, Sig-1R associates to heavy chain binding immunoglobulin protein (BiP, also known as glucose regulating protein 78, GRP78; or heat shock 70 kDa protein 5, HSPA5) in the ER (Hayashi and Su, 2007). BiP also translocates upon cell stress from the ER to the cell surface, exposes multiple domains on the cell surface and assumes new functions (Zhang et al., 2010), including virus recognition by its substrate-binding domain and facilitation of entry of several viruses, including CoV (Chu et al., 2018) (Figure 1). The capacity of BiP to facilitate surface attachment and viral entry likely depends on its binding to surface S (spike) viral proteins, as demonstrated for MERS-CoV and bat CoV-HKU9, and predicted for SARS-CoV-2 (Ibrahim et al., 2020). Sig-1R is engaged in protein trafficking from the ER to the plasma membrane, binds to BiP and, like BiP, it trasnlocates to the cell surface upon ER stress (Hayashi, 2019), but the involvement of Sig-1R in the export of BiP to the plasma membrane has not been investigated. Sig-1R antagonists inhibit Sig-1R-BiP dissociation at the ER (Hayashi and Su, 2007; Hayashi, 2019) and this might prevent BiP trafficking, surface expression and ultimately CoV attachment via BiP. Unlike Sig-1R, BiP is described as a non-membrane-bound ER lumenal chaperone. Thus, the interaction with Sig-1R could allow BiP stabilization/anchoring to the plasma membrane, although putative transmembrane domains have been identified allowing its potential, autonomous cell surface relocalization (Zhang et al., 2010). Yet, no direct interaction of Sig-1R with BiP has been specifically described at the plasma membrane. Similarly, no direct interaction with other host membrane proteins involved in viral attachment/entry (e.g., ACE2 or TMPRSS2) or with structural viral envelope proteins has been described substantiating Sig-1R-dependent modulation of viral entry. Alternatively, as discussed later, Sig-1R might regulate early stages of RNA replication and host cell response but not viral entry, whereas structural features shared by a number of sigma-1 ligands, independent on their binding to Sig-1R, might account for viral entry inhibition.

#### Sigma-1 Receptor Ligands as Lysosomotropic Agents?

The endocytic pathway (receptor-dependent endocytosis) is a basic mechanism for entry of CoV, including SARS-CoV, MERS-CoV and SARS-CoV-2, into host cells (Glebov, 2020; Yang and Shen, 2020). Binding of the spike (S) protein of SARS-CoV-2 to its receptor exposes its cleavage sites to cellular proteases, including endosomal acid proteases involved in endocytic processing (Millet and Whittaker, 2015; Pillay, 2020). In particular, endosomal cathepsin-mediated S protein cleavage is considered a critical step for CoV entry and initiation of infection (Millet and Whittaker, 2015; Glebov, 2020; Wędrowska et al., 2020; Yang and Shen, 2020).

Endosomes and maturation of endosomes into a lysosome is featured by their acidic internal pH, which is required for acid proteases and critical for SARS-CoV-2 processing and internalization (Wędrowska et al., 2020). The plasma and lysosomal membranes are highly permeable to the unionized form of weak bases but are essentially impervious to the protonated form of the bases (Marceau et al., 2012; Homolak and Kodvanj, 2020). Accordingly, weak bases, unionized in the cytoplasm, can cross the lysosomal membrane and enter the lysosome. Once in the lysosome, they are rapidly protonated since the lysosomal pH is considerably lower than the cytosolic pH, and become trapped inside lysosomes. This results in intralysosomal accumulation (ion trapping) of the drug and increased lysosomal pH (i.e., neutralization of lysosomal pH) sufficient to block most lysosomal enzymatic activity. If the concentration of the protonated base inside the lysosome is high enough, water enter the lysosome osmotically and the lysosomes swell to form large vacuoles (i.e., lysosomal vacuolation), with the consequent loss of lysosomal function (Aki et al., 2012).

Some drugs recognized as antiviral agents are lipophilic amines/weak bases that accumulate and preclude acidification of lysosomes, thus inhibiting virus internalization and post-internalization trafficking to the site of replication (Sieczkarski and Whittaker, 2002; Kaufmann and Krise, 2007; Mercer et al., 2010). Such lysosomotropism is shared by some lipophilic amines and cationic amphiphilic drugs (Kaufmann and Krise, 2007), including chloroquine and other anti-malarial drugs (Homolak and Kodvanj, 2020). Indeed, lysosome targeting agents are considered a potential therapy for COVID-19 (Homolak and Kodvanj, 2020). However, the effectiveness of this mechanism of action to control viral infections is hampered by its low specificity, cell compensation mechanisms to lower/restore intralysosomal pH and egress entrapped amines from lysosomes (Goldman et al., 2009), and the need for high drug dosage to allow substantial drug accumulation and alkalinization inside lysosomes, which also raises safety concerns.

A variety of marketed drugs fit within general physicochemical properties of lysosomotropic agents. Essentially, drugs with a ClogP > 2 and pKa between 6.5 and 11 can accumulate into lysosomes (Nadanaciva et al., 2011), although other physicochemical features also affect lysosomotropism (Kaufmann and Krise, 2007). Pharmacophore models for sigma-1 ligands (both putative agonists and antagonists) specify a positive ionizable group (i.e., a basic nitrogen, usually secondary or tertiary amine) flanked by hydrophobic regions (Pascual et al., 2019), which is coherent with potential lysosomotropism (Figure 1). This might have two implications. First, lysosomal sequestration might represent a barrier for a Sig-1R drug in reaching its intended target and would reduce its access to other cellular compartments (eg, ER, MAM or nuclear membranes) where Sig-1R is located for its antiviral effect to occur. Second, lysosomal trapping would result in unspecific, Sig-1R-independent defective acidification of lysosomes, and this off-target effect might be an added value for such drugs. It is clear that Sig-1R-mediated modulation of both viral replication and virus-induced ER-stress response might dependent on target-specific binding of ligands to Sig-1Rs in cellular compartments other than lysosomes, but it is presently unclear whether the pharmacophore-related, potential lysosomotropism of Sig-1R ligands actually hinders or contributes (and to what extent) to the activity reported for some Sig-1R ligands in antiviral drug screens.

### Sigma-1 Receptor Regulates Early Steps of Viral RNA Replication

In this section, evidence gained through gene silencing approaches are discussed. The antiviral activity exerted by numerous sigma-1 ligands in drug repurposing *in vitro* screens was not invariably unnoticed. Following the trail of pharmacological findings described before (Gastaminza et al., 2010), the role played by Sig-1R in HCV infection was investigated. RNAi though lentivirus-delivered short hairpin RNA (shRNA) targeting Sig-1R mRNA was used to downregulate Sig-1R expression in Huh-7 human hepatoma cells (Friesland et al., 2013). Four different shRNAs caused Sig-1R protein silencing with different magnitudes as compared with control cells transduced with an irrelevant shRNA. Control and silenced cells were inoculated with infectious HCV virions and infection efficiency was monitored by measuring the production of intracellular and extracellular progeny infectious virus as well as intracellular HCV RNA. Downregulation of Sig-1R expression in Huh-7 cells caused a proportional decrease in susceptibility to HCV infection, as shown by reduced HCV RNA accumulation and intra- and extracellular infectivity in single-cycle infection experiments. That is, progeny virus production was proportional to cellular Sig-1R levels at 24 and 48 h postinfection. Experiments were also conducted to explore the underlying mechanisms and revealed that Sig-1R downregulation did not affect HCV entry and that its expression levels were not limiting for primary translation of viral RNA genome, persistent HCV RNA replication (steady-state HCV RNA replication) or particle assembly and secretion. However, sigma-1 expression was rate limiting for launching HCV RNA replication. The reduced accumulation of HCV RNA in Sig-1R-deficient cells in single-cycle infection experiments was due to a defect in the establishment of HCV RNA replication, downstream of primary translation. Accordingly, Sig-1R expression is rate limiting for RNA replication early after primary translation but it is dispensable once the viral replication machinery has been established and replication reaches steady-state levels, as observed in persistently infected cells. Another remarkable result in the study by Friesland et al. (2013) is that Sig-1R expression in Huh-7 cells was rate limiting for HCV infection but not for infection with negative-sense single-stranded RNA viruses such as influenza A virus (A/WSN/33) or VSV (Friesland et al., 2013). Accordingly, evidence on the role played in viral replication by host Sig-1R in cultured hepatoma cells (not the primary cell target of SARS-CoV-2) infected with HCV (a +ssRNA virus, but not SARS-CoV-2) in no case imply proven mechanistic correlates against SARS-CoV-2 on its natural target cells.

Overall, data from Sig-1R deficient cells indicate that Sig-1R is a host cellular factor recruited for HCV infection, downstream entry, delivery and primary translation of viral RNA genome that regulates early stages of HCV RNA replication (Friesland et al., 2013). This is consistent with pharmacology findings whereby Sig-1R ligands (unrecognized as active ligands at Sig-1R in most studies) where found to inhibit early steps of the replicative cycle, after viral particle attachment, internalization and delivery of its genome to the cytosol (Nemerow and Cooper, 1984; Mingorance et al., 2014; Stadler et al., 2008; de Wilde et al., 2014; Gordon et al., 2020).

### Sigma-1 Receptor Colocalizes and Interacts with Non-Structural Proteins of the Viral Replicase/Transcriptase Complex

In this section, evidence gained from colocalization and interactome map studies are discussed. Sig-1R was found to colocalize with NS proteins of the HCV replication complex (Friesland et al., 2013). Cells were processed for double immunostaining with antibodies directed against components of the viral replicase (NS3, NS4B and NS5A) and against Sig-1Rs. In mock-infected Huh-7 cells, Sig-1R immunofluorescence revealed a predominant discrete cytoplasmic punctae localization that was juxtaposed to mitochondria as well as diffuse cytoplasmic pattern that colocalized with ER, the characteristic cellular distribution of Sig-1R in normal resting, unstressed cells (Su et al., 2010). During infection, the intracellular pattern of Sig-1R distribution changed: more that 70% of the infected cells displayed a diffuse perinuclear pattern 48 h postinfection. Interestingly, Sig-1R co-localized with viral NS3, NS4B and NS5A replicase components at perinuclear regions during early steps of viral infection. Later during infection (72 h), more than 60% of the infected cells displayed discrete cytoplasmic punctae that did not clearly colocalize with the bulk NS protein perinuclear signal, suggesting that a fraction of Sig-1R recovers the original pattern and that perinuclear colocalization of Sig-1R with viral replicase NS proteins observed at 48 h is transient. Overall, these results suggest that Sig-1R is recruited to perinuclear areas of the ER where NS proteins accumulate at early stages of viral infection to regulate the initiation of HCV RNA replication. Most Sig-1R and NS3 and NS5A were associated with detergent-resistant, cholesterol- and sphingolipid-rich intracellular membranes, further suggesting that Sig-1R and components of the HCV replicase target similar ER membrane environments, where Sig-1R likely exerts its proviral functions. Notably, such transient sigma-1 relocalization has been described during ER stress and proposed to contribute to the cellular response to stress (Hayashi and Su, 2007), suggesting that the virus takes advantage of host stress-related proteins to deploy a favorable cellular program. Cellular stress pathways induced by HCV infection to promote both viral replication and survival of the infected cell as well as the proviral role of Sig-1R in HCV infection have been reviewed (Vasallo and Gastaminza, 2015) and will not be reviewed further here.

Recently, a SARS-CoV-2 protein interaction map reveled a physical interaction with Sig-1R (Gordon et al., 2020). Authors cloned, tagged and expressed 26 of the 29 SARS-CoV-2 proteins and identified SARS-CoV-2-human protein-protein interactions using affinity-purification mass spectrometry. Approximately 40% of SARS-CoV-2 interacting proteins were associated with endomembrane compartments or vesicle trafficking pathways. In particular, the viral NS protein Nsp6 was specifically found to interact with Sig-1R. The SARS-CoV-2 genome encodes as many as 14 open reading frames (Orfs) (Masters, 2006; Chan et al., 2020; Gordon et al., 2020; Wu et al., 2020). The Orf1a/Orf1ab at the 5′ two-thirds of the genome encodes precursor polyproteins, which are auto-proteolytically processed into 16 NS proteins (Nsp1-16) that form the replicase/transcriptase complex. At the 3’ end of the viral genome, as many as 13 additional Orfs are expressed from sub-genomic mRNAs encoding Spike (S), Envelope (E), Membrane (M) and Nucleocapsid (N) structural proteins and putative accessory proteins. The viral replication machinery is thought to localize in ER membranes thanks to Nsp3, Nsp4 and Nsp6. Nsp6 forms complexes with Nsp3 and Nsp4 to anchor the viral replicase/transcriptase complex to ER membranes (Oostra et al., 2008; Alsaadi and Jones, 2019). All three replicase proteins contain transmembrane-spanning sequences important for assembly of the viral replicase/transcriptase complex to the ER membrane (Oostra et al., 2008). Nsp6 was shown to contain seven hydrophobic domains but six transmembrane domains, with its amino and carboxy termini exposed in the cytoplasm, and a conserved hydrophobic domain in the C-terminal cytosolic tail (Oostra et al., 2008; Baliji et al., 2009). Two nsp6 products of approximately 23 and 25 kDa were identified by Western immunoblotting, although the reason for the existence of multiple forms of nsp6 is currently unknown (Baliji et al., 2009). In addition to its role in anchoring the replicase complex to ER membranes, Nsp6 has been found to induce double-membrane vesicles and autophagosome formation (Cottam et al., 2011).

Positive-strand RNA viruses, including HCV and SARS-CoV, sequester host cell ER membranes to assemble viral replication. A network of modified perinuclear rough ER that integrates convoluted membranes, interconnected double-membrane vesicles and vesicle packets has been described (Gosert et al., 2002; Knoops et al., 2008; Sola et al., 2015). The viral replicase subunits were most abundantly located in convoluted ER membranes, RNA replication (double-stranded RNA) localized in double-membrane vesicles, and vesicle packets appeared to result from the merge of double-membrane vesicles and develop into large cytoplasmic vacuoles containing (budding) virus particles. Ultimately, replication of the CoV genome requires continuous RNA synthesis (Sola et al., 2015) and the reticulovesicular network provides a structural and functional continuum that connects ER membrane structures involved in RNA synthesis to sites at which the assembly of new virions occurs (Knoops et al., 2008). According to previous studies, Sig-1R is required at early stages of replication but not for steady-state HCV RNA replication or infectious particle assembly and secretion (Friesland et al., 2013). Thus, internalization, delivery and primary translation of the viral RNA genome would precede the recruitment of Sig-1R, which complexes with newly synthesized viral replicase proteins at initial stages before the reticulovesicular network continuum has fully developed in persistent infections. Early and transient colocalization of Sig-1R with HCV replicase proteins (Friesland et al., 2013) and interaction of Sig-1R with Nsp6 SARS-CoV-2 replicase protein (Gordon et al., 2020) support this hypothesis. The functional purpose of this interaction is unknown. A prompt assumption is that Sig-1R might assist insertion of the viral replication machinery to ER (convoluted) membranes, as anchoring of the replicase/transcriptase complex to the ER membrane is the proposed role of its partner Nsp6. However, it might also allow proper folding or membrane orientation of nascent viral proteins to assist multiprotein assembly of the functional replicase/transcriptase complex, promote early ER remodeling and trafficking through the reticulovesicular network, and/or regulate ER-mitochondrion signaling and ER-nucleus crosstalk to couple host cell bioenergetics and biosynthetic machinery to early viral demands. All these functions are coherent with the role played by this resident ER chaperone/scaffolding and dynamic pluripotent modulator protein, involved in inter-organelle signaling, bioenergetics and cellular stress responses (Hayashi and Su, 2007; Su et al., 2010; Vollrath et al., 2014; Hayashi, 2019; Delprat et al., 2020).

### A Role for Sigma-1 Receptor in Coronavirus-Induced Host Cellular Stress?

**FIGURE 2:**
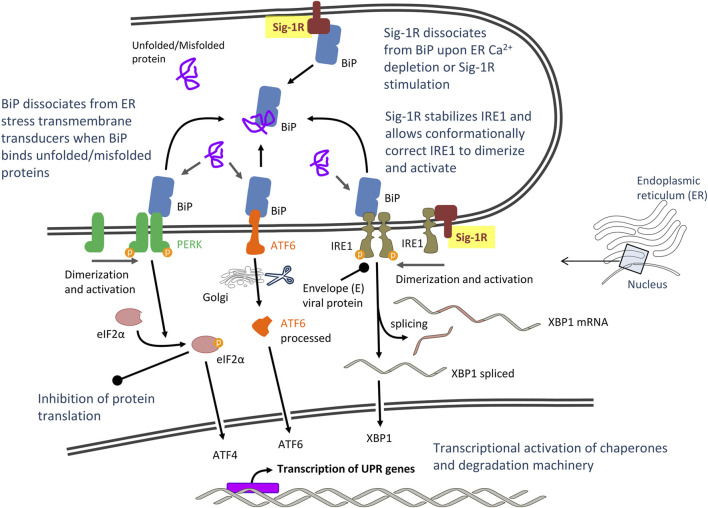
Proposed model of severe acute respiratory syndrome CoV-2 (SARS-CoV-2)-mediated unfolded protein response (UPR) signaling: Potential role of Sigma-1 receptors (Sig-1Rs) via interaction with master UPR regulators. Abbreviations and bibliographic references for the role of Sig-1R are provided in the text.

CoV infection of cultured cells causes ER stress and induces the unfolded protein response (UPR), the ER-specific stress response, and their downstream signals (Fung and Liu, 2014; Fung et al., 2014a). ER stress and UPR have been particularly involved in SARS-CoV-2 infection (Sureda et al., 2020) and combination therapies targeting COVID-19-mediated ER stress have been recently proposed (Banerjee et al., 2020). UPR aims to restore ER homeostasis and cell survival by global translation shutdown and increasing the ER folding capacity. The UPR signaling starts with the unfolded proteins activating three ER stress transducers: double-stranded RNA-activated protein kinase (PKR)-like ER protein kinase (PERK), activating transcriptional factor-6 (ATF6), or inositol-requiring enzyme (IRE1). Reversible dissociation from the ER lumenal chaperone BiP (also known as GRP78 or HSPA5) and interactions with other ER co-chaperones regulates the activation/deactivation dynamics of UPR transducers. BiP seems to be the direct ER stress sensor as it becomes activated by misfolded proteins. In unstressed cells, BiP binds to the ER lumenal domains of ER stress transducers and maintains them in an inactivated state (Figure 2). During ER stress, BiP preferentially binds to unfolded and misfolded proteins and dissociates from transmembrane transducers, facilitating their activation (Bertolotti et al., 2000; Kopp et al., 2019). Once activated, UPR transducers transmit the signal to the cytosol and the nucleus, and the cell responds by lowering the protein synthesis and increasing the ER folding capacity. The PERK/eIF2α (eukaryotic initiation factor 2α)/ATF4 pathway rapidly attenuates protein translation, whereas the ATF6 and the IRE1α/XBP1 (transcription factor X-box binding protein-1) cascades transcriptionally upregulate ER chaperone genes that promote proper folding (Figure 2). Accumulated unfolded proteins are either correctly refolded or unsuccessfully refolded and cleared via the ER associated degradation complex (ERAD) ubiquitin-proteasome pathway or via autophagy. However, under prolonged ER stress, UPR can also induce apoptotic cell death if homeostasis cannot be re-established and accumulation of misfolded protein becomes toxic. Apoptosis is triggered potentially via UPR-mediated and Ca^2+^-mediated caspase activation pathways and recruitment of mitochondria (Kim et al., 2006; Fung and Liu, 2014; Karagöz et al., 2019). Indeed, Ca^2+^ homeostasis plays a major role in ER stress and UPR-mediated apoptosis induction. Depletion of ER Ca^2+^ stores has detrimental effects on ER-resident Ca^2+^-dependent chaperones and protein folding, and undue Ca^2+^ transfer from ER to mitochondria at MAM (i.e., mitochondrial Ca^2+^ overload) leads to mitochondrial reactive oxygen species (ROS) production/oxidative stress and cytochrome C release (Carreras-Sureda et al., 2018). Finally, autophagy may also be activated under ER stress (ER stress-mediated autophagy) by pathways sharing common upstream signaling with UPR, including PERK, IRE1, ATF6 and Ca^2+^ (Song et al., 2017). Autophagy is characterized by the engulfment of cytoplasmic components in double-membrane-bound structures that are then delivered to lysosomes/vacuoles for degradation. Autophagosomes include worn-out proteins, protein aggregates and damaged organelles (Lee et al., 2015; Rashid et al., 2015; Song et al., 2017).

The burst of protein synthesis overloading the ER folding capacity, extensive rearrangement of the ER membrane during viral replication and viral proteins such as S (Siu et al., 2014) and 3a accessory (Minakshi et al., 2009) proteins of CoV cause ER stress, but viruses have evolved mechanisms to manage UPR signaling and create an environment favorable for its replication (Fung and Liu, 2014). Operative but hijacked UPR, with selective translational and transcriptional reprogramming but reduced susceptibility to cell death would contribute to host cell survival and sustain viral replication. Accordingly, CoV activate UPR transducers but induce minimal downstream induction of some UPR target genes. This favors a sustained shutdown of the synthesis of host cell proteins while the translation of viral proteins escalates (Bechill et al., 2008). Also favoring viral infection, the envelope E protein of SARS-CoV has been shown to neutralize the IRE1α/XBP1 pathway of UPR and inhibit apoptosis (DeDiego et al., 2011). Note that apoptosis is a fatal fate for the infected cell, but it protects the host by limiting virus production and dissemination. However, not all the evidence has the same directionality and some findings support that ER stress, UPR and autophagy induction are innate responses in cell host’s struggle with CoV. For instance, infection with the alphaCoV transmissible gastroenteritis virus (TGEV) activated all three UPR pathways (PERK, ATF6 and IRE1), but activation of the PERK/eIF2α axis inhibited TGEV replication through overall attenuation of protein translation (Xue et al., 2018). The PERK pathway was also activated in cells expressing the 3a accessory protein of SARS-CoV, a protein that is pro-apoptotic (Minakshi et al., 2009). Other studies point to a mix of positive and negative effects on viral replication. For instance, IRE1 RNase activity was reported to be unfavorable to viral replication whereas IRE1 kinase activity enhanced it (Su et al., 2017).

What about Sig-1R? ER stress/UPR induces Sig-1R expression through the PERK/eIF2α/ATF4 pathway (ATF4 binds to the 5' flanking region of Sig-1R gene to upregulate its transcription) (Mitsuda et al., 2011). In turn, Sig-1R upregulation, experimental overexpression or its ligand stimulation protects cells, which correlates with reduced ER stress and apoptosis in most studies (Mitsuda et al., 2011; Wang et al., 2012; Omi et al., 2014; Shimazawa et al., 2015; Cao et al., 2017; Ellis et al., 2017; Morihara et al., 2018; Zhai et al., 2019), but not all (Penas et al., 2011; Schrock et al., 2013; Alam et al., 2017). A biphasic role has also been described, with Sig-1R-mediated exacerbation followed by protection, concomitant with increased and reduced markers of ER stress and autophagy response, respectively (Yang et al., 2017). In paragraphs below, evidence supporting a role of Sig-1R in modulating several aspects of the ER stress response potentially relevant for CoV infection is reviewed and discussed.

#### Endomembrane Remodeling

ER remodeling is a key early element of ER stress response induced by CoVs. As discussed before, CoVs benefit from endomembrane compartments and induce the growth and remodeling of host cell ER membranes to form a reticulovesicular network (Knoops et al., 2008). Depletion of Sig-1R leads to abnormal ER morphology including loss of ER tethering and proliferation as well as mitochondrial abnormalities and mitophagy, suggesting a role of Sig-1R in maintaining structural and functional integrity of the ER and mitochondria (Vollrath et al., 2014). Thus, pharmacological blocking of Sig-1R might hinder ER remodeling and challenge mitochondrial energy supply, both required for viral replication. This is coherent with the finding that Sig-1R is required at early stages of HCV replication (Friesland et al., 2013), when ER remodeling and anchoring of the viral replicase complex occurs. Unfortunately, the role played by Sig-1R in architectonics of ER membranes during viral infection has not been investigated.

#### Calcium Homeostasis

**FIGURE 3:**
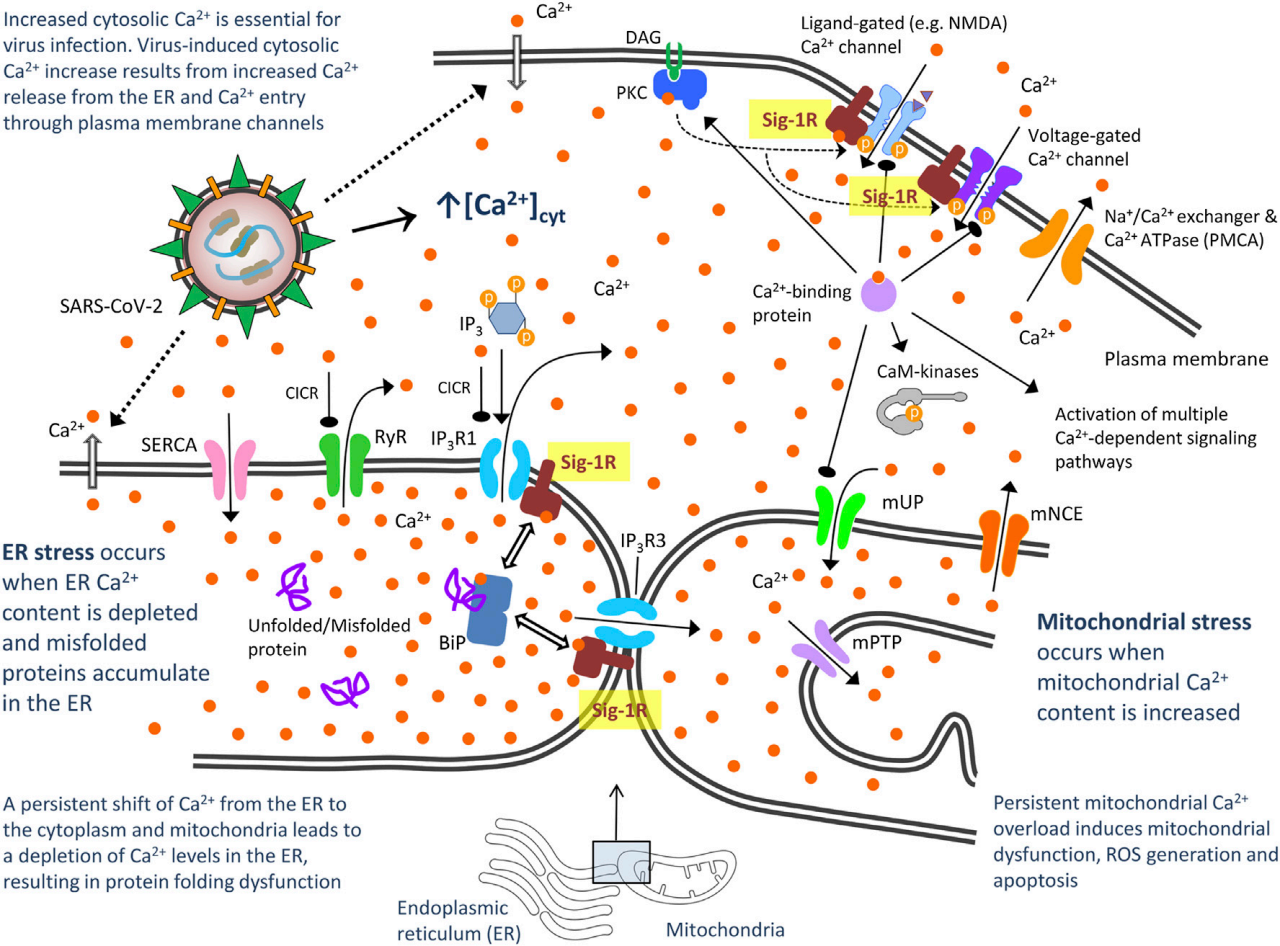
Proposed model of severe acute respiratory syndrome CoV-2 (SARS-CoV-2)-mediated disturbance of host cell calcium (Ca^2+^) homeostasis: Potential role of Sigma-1 receptors via interaction with Ca^2+^ channels in the plasma membrane and ER. CaM kinases, Ca^2+^/calmodulin-dependent protein kinases; DAG, diacylglycerol; IP_3_, inositol 1,4,5-triphosphate; IP_3_Rs, inositol 1,4,5-triphosphate receptor-gated channels; IP_3_R1 mediates ca^2+^ release from ER to cytosol and IP_3_R3 to mitochondria. They are activated not only by IP_3_, but also by low Ca^2+^ concentrations through a process often referred to as Ca^2+^-induced Ca^2+^ release (CICR). High cytosolic Ca^2+^ concentration can instead inhibit IP_3_Rs; PKC, protein kinase C; mNCE, mitocondral Na^+^/Ca^2+^ and 2H^+^/Ca^2+^ exchangers. They slowly eject accumulated Ca^2+^ back into the cytosol; mPTP, mitochondrial permeability transition pore. Once intramitochondrial Ca^2+^ rises above a certain threshold, this voltage- and Ca^2+^-dependent high-conductance channel in the inner membrane opens, activating cell death mechanisms; mUP, mitochondrial uniporter. It is gated by Ca^2+^ in a biphasic manner. Ca^2+^ uptake into mitochondria is facilitated by Ca^2+^/calmodulin, but sustained cytosolic Ca^2+^ levels inactivate the uniporter, preventing further Ca^2+^ uptake; RyR, ryanodine receptor. It is a Ca^2+^-gated Ca^2+^ channels (CICR); SERCA, sarco/endoplasmic reticulum Ca^2+^ ATPase. It restores ER with Ca^2+^. Bibliographic references for the role of Sig-1R are provided in the text.

Viruses have evolved mechanisms to disturb host cell Ca^2+^ homeostasis and increase intracellular Ca^2+^ as Ca^2+^ is essential for virus entry, replication, maturation and release (Olivier, 1996; Chen et al., 2019). Impeding virus-induced abnormal cytosolic Ca^2+^ increase by blocking Ca^2+^ release from the ER or Ca^2+^ entry through plasma membrane channels/pumps has emerged as a strategy to control viral infections (Chen et al., 2019). Accordingly, some Ca^2+^ channel blockers have been reported to improve mortality and decrease risk for intubation and mechanical ventilation in elderly patients hospitalized for COVID-19 (Solaimanzadeh, 2020). The Sig-1R regulates both Ca^2+^ entry at the plasma membrane level (via interaction with ligand- and voltage-gated Ca^2+^ channels) and Ca^2+^ mobilization from endoplasmic stores [via interaction with inositol-1,4,5 trisphosphate receptors, (IP_3_Rs)] (Monnet, 2005). Under ER stress (Ca^2+^ depletion from ER stores), Sig-1R dissociates from BiP and chaperones IP_3_R3, ensuring proper Ca^2+^ signaling from the ER into mitochondria (Hayashi and Su, 2001; Wu and Bowen, 2008) (Figure 3). Increased IP_3_R3-mediated Ca^2+^ flow to mitochondria at MAM is fundamental for coupling cell physiology to energy demand, which is likely required for virus protein anabolism and RNA synthesis, but sustained/excessive Ca^2+^ influx into mitochondria results in excessive ROS, oxidative stress and apoptosis. Sig-1R agonists cause dissociation of Sig-1R from BiP, allow Sig-1R- IP_3_R3 interaction and thus enhance IP_3_R3-mediated Ca^2+^ flow to mitochondria whereas Sig-1R antagonists do not affect the Sig-1R-BiP association but inhibit the dissociation mediated by Sig-1R agonists. A Ying-Yang effect has been described for Sig-1R agonists, by increasing mitochondrial complex I activity and triggering moderate ROS increase in a Ca^2+^-dependent manner as a physiological signal, but attenuating complex I and IV dysfunctions and promoting a marked anti-oxidant effect in pathological conditions (Goguadze et al., 2019). Treatment of mitochondrial membranes with the Sig-1R agonist (+)-pentazocine leads to phosphorylation of Bad and NADPH-dependent production of ROS through Rac1 signaling (Natsvlishvili et al., 2015). Immunoprecipitation techniques revealed that Sig-1R at MAM form complexes with Rac1, IP_3_R and Bcl2, and Sig-1R agonists could induce mild oxidative stress through this IP_3_R/Sig-1R/Bcl2/Rac1 multiprotein complex (Natsvlishvili et al., 2015). Altogether, both bioenergetic coupling and mitochondrial Ca^2+^ overflow-mediated apoptosis are dependent on Ca^2+^ signaling through IP_3_R3 at MAM and are regulated by Sig-1R (Delprat et al., 2020). Ca^2+^ release from ER to cytosol via increased IP_3_R1 activity also induces ER stress and Sig-1R binds to and regulates IP_3_R1s as well (Kubickova et al., 2018) (Figure 3). Fine tune control (enough for enhanced energy supply but not too much to avoid host cell death) of these mechanisms might be essential for efficient viral infection, thus suggesting that pharmacological modulation of Sig-1R offers here a therapeutic opportunity to counteract the virus program (Figure 3).

#### Intreraction with Master Unfolded Protein Response Regulators

Sig-1R binds in a dynamic, reversible and Ca^2+^-dependent manner to the ER lumenal chaperone and stress sensor BiP (Hayashi and Su, 2007; Ortega-Roldan et al., 2013). BiP, also referred to as GRP78, is an important host factor for viral infection. A substantial amount of SARS-CoV S protein accumulates in the ER during infection and induces direct activation of BiP and UPR selective pathways (Chan et al., 2006). Targeting BiP has the potential to disrupt multiple stages of the viral life and it has recently proposed as a potential therapeutic approach for CoV infection (Ha et al., 2020). Sig-1R binds the nucleotide-binding domain of BiP though its bulky C-terminal lumenal domain (Ortega-Roldan et al., 2013). Dissociation of ER membrane-bound Sig-1R from lumenal BiP occurs upon Ca^2+^ depletion (indicative of ER stress) or pharmacological Sig-1R stimulation (Figure 2). BiP also binds to the ER lumenal domains of membrane-bound UPR transducers PERK and IRE1 and, when bounded, both BiP and UPR transducers remain in an inactive state. Recruitment of misfolded proteins to BiP substrate-binding domain during ER stress stimulates ATPase activity within its nucleotide-binding domain, enabling BiP to adopt an ADP-bound conformation that dissociates from PERK and IRE1 to allow their activation and initiation of UPR signaling cascades (Bertolotti et al., 2000; Kopp et al., 2019). The mechanistic details for Sig-1R modulation of UPR via interaction with BiP are unknown. Does Sig-1R-BiP dissociation act as a co-activator (together with misfolded proteins and ATP binding) to induce UPR? Does Sig-1R act as an allosteric inductor or compete with PERK and/or IRE1 for binding to the BiP nucleotide-binding domain? Despite these and other unanswered questions, evidence places Sig-1R as a sensor of ER stress (Ca^2+^ depletion) and upstream regulator of UPR. Does it support the antiviral effect of Sig-1R ligands in numerous cellular assays?

In addition to its interaction with its co-chaperone BiP, Sig-1R also chaperones the ER resident transmembrane protein IRE1 (Mori et al., 2013), one of the ER stress transducers, important for CoVs to adapt host cellular machinery to their demands and antagonize cell apoptosis (Fung et al., 2014b). Sig-1R stabilizes IRE1 when cells are under ER stress and such interaction allows conformationally correct IRE1 to dimerize to the activated form (Mori et al., 2013) (Figure 2). IRE1 (alpha isoform, IRE1α) has RNase activity coupled to kinase activity. There are different models proposed for IRE1 activation and all of them involve dissociation from BiP, oligomerization and activation of its cytosolic kinase domain (Adams et al., 2019). This activation allows unconventional splicing of XBP1 mRNA and subsequent translation of an active transcription factor, XBP1s. XBP1s promotes expression of several targets including chaperones, foldases and components of the ERAD pathway in order to restore protein homeostasis (Smith et al., 2011). The envelope E protein of SARS-CoV has been shown to counteract the IRE1/XBP1 pathway of UPR (DeDiego et al., 2011), suggesting that inhibition of the IRE1/XBP1 pathway of UPR is important for CoV infection. Studies performed on the herpes simplex virus-1 replication showed an opposite action of IRE1 domains on viral replication, RNase activity being unfavorable to viral replication and kinase activity enhancing it (Su et al., 2017). IRE1 RNase activity activates the cellular protein degradation pathway (ERAD) that might lead to the degradation of viral proteins, which is unfavorable to viral replication (Su et al., 2017). Accordingly, in order to facilitate viral replication, IRE1 RNase activity was suppressed in infections by a variety of viruses, including CoV mouse hepatitis virus (MHV) (Bechill et al., 2008). The RNase activity of IRE1 may also target other genes via regulated IRE1-dependent decay (RIDD). RIDD is the mechanism by which IRE1 cleaves target transcript substrates that are degraded and contributes to the maintenance of ER homeostasis by diminishing ER protein load via mRNA degradation, but it has also been proposed to lead to cell death (Tam et al., 2014; Abdullah and Ravanan, 2018). Sig-1R associates to and restricts IRE1 endonuclease (RNAase) activity, needed for splicing the mRNA encoding XBP1 to produce active XBP1 protein in preclinical models of sepsis and inflammation (Rosen et al., 2019). Indeed, LPS-challenged Sig-1R knockout mice had increased hepatic XBP1 splicing when compared to WT mice. The mechanism by which the virus impairs IRE1 RNase activity is unknown, but pharmacological blocking of Sig-1R might promote IRE1 RNase activity and thus increase IRE1/XBP1-dependent degradation pathways. Is it contributing to the inhibitory effect of Sig-1R antagonist ligands on viral replication?

#### Autophagy

Finally, CoV infection (inclusive of SARS-CoV, MERS-CoV and the new SARS-CoV-2) has been demonstrated to induce autophagy (Maier and Britton, 2012; Yang and Shen, 2020). Interestingly, expression of viral Nsp6 from diverse CoVs induces autophagy (Cottam et al., 2011). Viral replication proteins from MHV and SARS CoVs have been shown to colocalize with autophagosome protein markers (Prentice et al., 2004a; Prentice et al., 2004b) and autophagy has been implicated in both the formation of double-membrane vesicles and replication of MHV (Prentice et al., 2004a). However, colocalization of autophagosome markers with specific replicase subunits of SARS-CoV was not observed in other study (Snijder et al., 2006) and a number of observations suggest that autophagy is not directly implicated in viral replication (Zhao et al., 2007). On the contrary, it was reported that MERS-CoV multiplication exerted an inhibitory effect on the autophagy process and that enhancement of autophagy reduced the replication of MERS-CoV (Gassen et al., 2019). Thus, it is controversial whether autophagy is used by viruses in their benefit or whether it actually represents a protective cellular response against CoV infections. Autophagosomes originate from the ER-mitochondria contact site (Hamasaki et al., 2013) and Sig-1R acts at this MAM intersection as an upstream modulator of autophagy (Schrock et al., 2013). Sig-1R agonists trigger autophagy after extended treatment, whereas Sig-1R antagonists and knockdown of Sig-1R suppresses autophagome formation (Schrock et al., 2013). Accordingly, loss-of-function mutations and Sig-1R deficiency are associated with defective autophagy, leading to accumulation of autophagic vacuoles. In contrast, re-expressing Sig-1R in the null background or its activation restores/induces autophagic activity (Vollrath et al., 2014; MacVicar et al., 2015; Christ et al., 2019; Yang et al., 2019; Christ et al., 2020). Sig-1R is not likely a core component of the general physiological autophagy machinery but it seems needed for cellular stress-induced autophagy (MacVicar et al., 2015). Despite the known interaction of transmembrane SARS-CoV-2 Nsp6 with host Sig-1R protein, the induction of autophagy by Nsp6 and the role played by Sig-1R in autophagy regulation, it is uncertain whether and how Sig-1R is implicated in autophagy induction secondary to CoV infection.

## Author Contributions

The author confirms being the sole contributor of this work and has approved it for publication.

## Conflict of Interest

JV was a full-time employee in ESTEVE PHARMACEUTICAS at the time of review.
